# Practices for Alleviating Heat Stress of Dairy Cows in Humid Continental Climates: A Literature Review

**DOI:** 10.3390/ani7050037

**Published:** 2017-05-02

**Authors:** Sébastien Fournel, Véronique Ouellet, Édith Charbonneau

**Affiliations:** Département des sciences animales, Université Laval, 2425 rue de l’Agriculture, Québec City, QC G1V 0A6, Canada; veronique.ouellet.6@ulaval.ca (V.O.); edith.charbonneau@fsaa.ulaval.ca (E.C.)

**Keywords:** dairy cows, heat stress, cooling systems, performance, health, temperature-humidity index

## Abstract

**Simple Summary:**

The severity of heat stress issues on dairy cows will increase as global warming progresses. Fortunately, major advances in environmental management, including fans, misters, sprinklers, and cooled waterbeds, can attenuate the effects of thermal stress on cow health, production, and reproduction. These cooling systems were, however, tested in subtropical areas and their efficiency in northern regions is uncertain. This article assesses the potential of existing technologies to cool cows in humid continental climates through calculation of heat stress indices.

**Abstract:**

Heat stress negatively affects the health and performance of dairy cows, resulting in considerable economic losses for the industry. In future years, climate change will exacerbate these losses by making the climate warmer. Physical modification of the environment is considered to be the primary means of reducing adverse effects of hot weather conditions. At present, to reduce stressful heat exposure and to cool cows, dairy farms rely on shade screens and various forms of forced convection and evaporative cooling that may include fans and misters, feed-line sprinklers, and tunnel- or cross-ventilated buildings. However, these systems have been mainly tested in subtropical areas and thus their efficiency in humid continental climates, such as in the province of Québec, Canada, is unclear. Therefore, this study reviewed the available cooling applications and assessed their potential for northern regions. Thermal stress indices such as the temperature-humidity index (THI) were used to evaluate the different cooling strategies.

## 1. Introduction

The physiological (e.g., respiration rate) and behavioural (e.g., resting pattern) controls of dairy cows attempt to maintain a constant body temperature by regulating their thermal energy balance, so that heat input through metabolism (maintenance, exercise, growth, lactation, gestation, and feeding) equals heat loss to the environment (by conduction, convection, and evaporation). When environmental conditions exceed a threshold limit that increases the core-body temperature, heat stress sets in and animal welfare can be compromised. Moreover, cow health, production, and reproduction performance are reduced under heat stress [1,2].

Feed intake begins to decline at air temperatures of 25–26 °C in lactating cows [3] as reducing dry matter ingestion is a way to decrease heat production in warm environments [4]. More specifically, the reduction of dry matter intake for heat-stressed cows is about 10 to 15% relative to cooled cows [5]. Cows under heat stress also have elevated respiration and sweating rates, which results in greater body fluid losses that increase maintenance requirements to control dehydration and blood homeostasis [4]. For high-producing cows, Berman et al. [6] found that the respiratory frequency started rising above 50–60 breaths min^−1^ at ambient temperatures higher than 25 °C. Other studies showed a heat-induced decline in milk yield, which can reach 14% during early lactation and 35% during mid-lactation [7]. Reproduction is affected by high temperature because it reduces the expression of oestrous behaviour, modifies follicular growth, and inhibits embryonic development. As a result of the effects of thermal stress, the dairy industry experiences significant financial losses every year, with annual estimates between $900 and $1500 million in the United States (US) [8].

A broad spectrum of strategies can be used to temper the impact of a hot climate on animal production and dairy profitability, but physical modification of the environment is the primary means. The typical methods can be divided into two groups: (i) those modifying the environment to prevent or limit the degree of heat stress to which cows are exposed; or (ii) those enhancing heat exchange between cows and their environment. Practically, this means increasing the evaporative cooling rate by wetting cows or the air around them (e.g., misters and sprinklers) and increasing convective heat transfer rate by increasing air speed over cows (e.g., fans) [9,10].

Much of the research on cooling systems has been carried out in subtropical regions (e.g., Southern US, Israel, and Saudi Arabia) under conditions of moderate to severe heat stress for durations of several months (typically >30 °C and 40% relative humidity). Although episodic mild to moderate heat stress (typically 25 °C and 75% relative humidity) that occurs under humid continental climates (e.g., Eastern Canada and Northern US) has also been recognised as problematic [11,12], there is limited information available regarding the efficiency of physical applications to abate thermal stress in these conditions. Nevertheless, representative hot environments in both climate zones nearly correspond to the same value (75–77) on a temperature-humidity index (THI) chart [1]. Moreover, in the context of climate change where mean annual temperature is expected to rise by 2–4 °C by 2050 in humid continental areas like the province of Québec (Canada) [13], heat mitigation systems should become more and more popular. Therefore, a better understanding of the potential for cooling options to reduce heat stress on northern dairy farms from a global warming perspective may be relevant.

The evaluation and comparison of the different available practices in this paper are based on thermal stress indices that estimate the impact of ambient conditions around animals on their performance and comfort. The THI has been widely used for this purpose [2,14]. By capturing much of the heat exchange impact of warm environments through temperature and humidity, it often adequately represents the overall effect on livestock [1]. Numerous studies established THI thresholds for heat stress in cattle: comfort (THI < 68), mild discomfort (68 < THI < 72), discomfort (72 < THI < 75), alert (75 < THI < 79), danger (79 < THI < 84), and emergency (THI > 84) [15]. Other indices like the black globe-humidity index (BGHI) and the heat load index (HLI) that add the impact of solar radiation and convective cooling of wind (limitations of the THI) can also be used for certain situations [16]. It is then possible with these indicators to assess the efficiency of cooling systems used under different climates (subtropical versus humid continental).

## 2. Methods for Reducing Heat Stress

The environmental modifications presented in this section attempt to reduce the potential for heat stress by lowering the solar radiation or temperature around the animal.

### 2.1. Shading Systems

The first step that should be taken to moderate the stressful effects of a hot climate is to protect cows from direct solar radiation. Shading, either natural or artificial, is one of the most easily implemented and economical methods of minimising heat from solar radiation, but does not alter air temperature nor relative humidity around cows to maximise sensible routes of heat loss [9,17,18].

#### 2.1.1. Shade for Outside Lots

It has been estimated that total heat load in outside lots can be reduced by 30% or more with a well-designed shade [19,20]. Trees can be very effective for providing shade to animals [2,3,9,21,22] as black globe temperature, which integrates effects of net radiation, dry-bulb temperature, and wind speed, measured under two tree shades (29.0–30.2 °C) was significantly lower than that recorded outside the shade (35.5 °C). In addition, rectal temperatures (39.3 and 40.1 °C, respectively) and respiration rates (61 and 79 breaths min^−1^, respectively) for shaded versus unshaded lactating cows were significantly decreased [23].

Artificial shades (e.g., cloth roof on metal or wood frame with wheels or skids and gable roof structure) have also been used with success since they significantly reduce black globe temperature. Several studies [24,25,26,27,28,29,30,31] reported declines in black globe temperature between 2 and 11 °C under a shade compared with the outside. Consequently, climate differences resulted in variation of thermal stress indicators such as BGHI (4 units) and HLI (13 units) for shaded and unshaded environments (Table 1). The THI values did not differ between both systems since THI does not fully predict heat stress if convective cooling is increased and if solar radiation is reduced under shades [32].

A shaded environment also impacts physiological responses of lactating dairy cows (Table 1). Research works [29,30,33,34] have found an average drop of 0.2 °C of vaginal temperatures between shaded and unshaded cows. Other studies [24,26,35] showed that cows with access to shade had lower rectal temperatures than those without (38.7 and 39.3 °C, respectively). In fact, shade allowed cow rectal temperatures to fall below 39 °C, the level at which heat stress is sufficient to affect milk yield and fertility [3]. Shaded cows generally produce 0.7 kg∙day^−1^ more milk than unshaded cows [25,26,29,30,36]. The conception rate can be increased by the use of shade, passing from 25.3% in an unshaded situation to 44.4% in a shaded situation [26]. Significant respiration rate decreases (8–36 breaths min^−1^) were observed for shaded cows during hot summer days [24,26,33,35]. In most cases, shade allowed respiratory frequency to pass under the critical threshold of 60 breaths min^−1^ [3].

#### 2.1.2. Shade for Dairy Barns

Preventing excess solar heat from entering the building can help to reduce heat load on the animal. The first design criteria to consider should be the orientation of the structure. For example, naturally-ventilated barns with north-to-south orientations have greater solar radiation exposures than barns with east-to-west orientations since sunlight can directly enter both in the morning and afternoon [2]. A trial in California showed an increase in morning (4 breaths min^−1^) and afternoon (9 breaths min^−1^) respiration rates of cows when barns were orientated north-to-south versus east-to-west [37]. Otherwise, appropriate window glazing and fixed or seasonal shading options, including overhangs, awnings, and trees, may be used to avoid or reduce the need for cooling [38].

### 2.2. Roof Insulation

A good thermal insulation of the barn roof is another technique that physically modifies the barn environment and that can limit the adverse effects of high ambient temperature [39]. In warm weather, insulation reduces the flow of heat into the barn and keeps it cooler [10,40]. Indeed, it was found that the addition of insulation beneath the roof of an open shelter reduced dry-bulb temperature and black globe temperature by 1.2 and 2.0 °C, respectively (Table 2). Similarly, THI and BGHI were lessened by 1.3 and 2.2 units, respectively. Consequently, animals in the insulated area consumed more feed (0.2 kg∙day^−1^) and produced more milk (0.6 kg∙day^−1^). However, differences were not significant for body temperature and respiration rate [41,42]. A combination of insulated shade and sprinklers was tested by Fuquay et al. [42]. This type of environmental modification improved cow comfort as indicated by lower rectal temperatures (38.7 and 38.8 °C, respectively) and respiration rates (71 and 78 breaths min^−1^, respectively) compared with a control treatment.

### 2.3. Air Cooling Systems

#### 2.3.1. Air Conditioning and Zone Cooling

Air conditioning is the most effective option for reducing air temperature and relative humidity. In Arizona, Wiersma and Stott [47] and Stott and Wiersma [48] maintained the conditions inside a refrigerated dairy barn at a THI of 71 and found that fertility was improved. Other studies [49,50,51] reported a significant increase in 4% fat-corrected milk yield in Florida and Louisiana when cows were air-conditioned compared with cows kept outside. In Missouri, Hahn et al. [52] compared an air-conditioned barn with a dry lot under naturally varying conditions. The cooling system, which provided better environmental conditions (Table 3), resulted in an average reduction in rectal temperature (0.4 °C) and a net increase in milk yield (0.5 kg∙cow^−1^∙day^−1^). Recently, environmental conditions in an air-conditioned barn and in evaporatively cooled sprinkler and fan and tunnel-ventilated buildings were compared by Bucklin et al. [32]. All cooling methods generally provided conditions more comfortable in the facilities than those outside. However, only the air-conditioned barn maintained the THI consistently below 72 (Table 3).

Zone cooling or inspired-air cooling applies a jet of cooled air onto the head and neck of animals [17,38]. Early studies conducted by Kleiber and Regan [53] and Hahn [54] indicated that cooling the inspired air (19.4–27.8 °C below ambient air) for lactating Holstein cows while they were subjected to a hot environment (32.2–40.5 °C) decreased rectal temperature and respiratory frequency. Similarly, Hahn et al. [55] and Canton et al. [31] found effective reduction of rectal temperature and respiration rate with inspired-air treatments of 8.5–15.5 °C. Roussel and Beatty [56], Fuquay et al. [42], and Gomila et al. [57] also reported significantly lower rectal temperatures and respiration rates for zone-cooled cows. Roussel and Beatty [56] and Gomila et al. [57] reported 7 to 19% increases in milk production for cows provided with inspired-air cooling treatments (Table 4).

#### 2.3.2. Foggers and Misters

Evaporative cooling systems use the energy from the air to evaporate water. The evaporation of water into warm air reduces the air temperature while increasing relative humidity [9,38]. Water can be evaporated from atomizing nozzles (next paragraphs) or cooling pads (Section 2.3.3). The first group includes foggers and misters.

High-pressure (>200 psi) fogging systems integrating a ring of fogging nozzles to circulation fans disperse very fine droplets of water into the surrounding air. As fog droplets are emitted, they are immediately spread into the fan’s air stream where they soon evaporate. Animals are chilled as cooled air is blown over their bodies and as they inspire cooled air [10,22,58,59]. Misting systems generate larger droplets (15 and 50 µm in diameter) than fogging systems, but cool the air by the same principle [9,22,38,58,60].

Bucklin et al. [32] tested a fogging system in a tunnel-ventilated barn in Florida. It also contained feed faced sprinklers that worked only at night when foggers were turned off. The environmental conditions of this building were compared with those outside and in another tunnel barn with only 24 h day^−1^ sprinklers (Table 5). The THI in the barn with foggers was slightly lower compared with the other treatments, resulting in lower body temperatures of cows. Brouk et al. [61] reported similar results for observations collected in the same two barns during the same time period.

Misting systems were used with good success in the US (Arizona, Kansas, and California) and Saudi Arabia where humidity is typically low [62,63,64,65,66]. Numerous studies [67,68,69,70,71,72,73,74,75] compared the environmental conditions and physiological responses of dairy cows under misters with those obtained under an open-sided barn alone or equipped with fans (Table 6). Globally, ambient temperature in the facilities using misting technologies was 2–9 °C cooler than the barns without evaporative cooling, while relative humidity was increased by 8–50 units of percentage with misters. Overall, misting systems reduced THI by 1 to 5 units [66,67,71,73]. This is in accordance with the formula developed by St-Pierre et al. [8] from published data that quantified similar declines in THI for misting systems used in hot temperatures. Dairy cows generally benefitted from misters since rectal temperatures (−0.8 °C) and respiration rates (−20 breaths min^−1^) were reduced when used [66,67,68,70,71,72,73,74]. Only Chan et al. [69] observed no significant effect of misters on these parameters. Several research works [68,71,72,73,75] concluded that misting systems positively affects milk yield (+1.7 kg∙day^−1^).

#### 2.3.3. Evaporative Cooling Pads

Evaporative cooling pads made of fibrous material woven together with large gaps in the grooves are generally added to the air inlets of tunnel-ventilated barns (Section 3.1.3). In this way, the incoming air is pulled through a saturated medium where the conversion of water from a liquid to a vapour phase removes heat energy from the incoming air, which lowers its temperature but increases its relative humidity. Cooling efficiency is about 55–75% for most evaporative cooling pads, but these water-based systems are prone to plugging and algae growth [22,76].

Tunnel-ventilated, water-padded barns reportedly lower room temperature during the hottest part of the day by 2.3–5.6 °C (Table 7). However, because relative humidity is increased (22–27 units of percentage), the impact of combined tunnel ventilation and cooling pads on THI is usually minor (2 units). The installation of wetting pads in a housing facility may reduce core-body temperature (up to 1.0 °C) and respiration rate (2–22 breaths min^−1^) when compared with open-sided barns without cooling systems or with fans and/or sprinklers [77,78,79,80,81,82]. The effect was slightly different for Shiao et al. [83], with vaginal temperature and respiration rate mostly affected by the sprinkling system. Finally, pads had some or no effect on dry matter intake and milk yield (Table 7).

## 3. Methods for Enhancing Cow Heat Losses

Increased heat exchange generally involves increasing heat loss from the body surface by enhancing heat loss mechanisms that include conduction (direct contact with a surface), convection (contact with a moving fluid), and evaporation (liquid-to-vapour phase change for water through the respiratory system and skin).

### 3.1. Air Movement

Air movement is an important factor in the relief of heat stress, as it affects both convective and evaporative heat losses. Additional circulation fans can be installed in the barns if airflow provided by natural or mechanical ventilation is not sufficient [9].

#### 3.1.1. Panel or Basket Fans

Panel or basket fans are usually selected for cooling. Typically, these low volume (235–470 L∙s^−1^), high speed (1.0–2.5 m∙s^−1^) fans (LVHS) are 0.6–1.2 m in diameter. They should be spaced at a distance of 10 times their diameter, positioned so they blow air in the direction of the prevailing winds, and tilted downwards to aim their airflow at a point directly below the next fan in line [2,10,84,85].

The addition of LVHS fans to open-sided barns benefitted dairy cows (Table 8). Indeed, ventilated cows had a lower rectal temperature (−0.4 °C) and respiration rate (−11 breaths min^−1^) and a higher conception rate (+30 units of percentage) compared with control cows. The former also ate more feed (+0.6 kg∙day^−1^) and produced 1 kg∙day^−1^ more milk.

#### 3.1.2. Big Ceiling Fans

An alternative to panel or basket fans for hot weather ventilation is high volume, low speed (HVLS) fans. Typically, 2.4–7.5 m diameter ceiling fans are installed 12–18 m apart, along the length of a barn. Fans run at 50 rpm and move 50,000–200,000 L∙s^−1^ of air [2,10,38,85,88,89].

Bucklin et al. [32] experimented with ceiling fans inside an open-sided barn equipped with sprinklers. The conditions within the building were greatly improved (−4.7 THI units) compared with those outside (Table 9). This result is in accordance with the formula developed by St-Pierre et al. [8] from published data which quantifies similar declines in THI for a system of fans used during periods of heat stress. Worley and Bernard [90] compared air movement patterns, wind speeds, and cow vaginal temperature for HVLS fans with a combination of LVHS fans and misters in a typical dairy free-stall barn. While air speed was more evenly distributed throughout the barn with the HVLS system, the ceiling fans did not provide the desired air speed within the critical areas of the barn. For the extremely hot, humid conditions considered during the experiment, internal temperatures for both groups of cows were above normal, indicating heat stress, but they were higher (0.2 °C) for cows cooled by the HVLS system than for those cooled by circulation fans. Meyer et al. [91] also observed better results in terms of respiration rate (−9 breaths min^−1^) and milk yield (+3 kg**∙**day^−1^) with LVHS than HVLS fans.

#### 3.1.3. Tunnel Ventilation

Tunnel ventilation is another option that increases airflow to reduce heat stress. With a tunnel ventilation system, large fans (1.2–1.8 m in diameter) are used to move air through a barn at an air speed (1–3 m∙s^−1^) fast enough to provide a beneficial wind chill effect that cools the cows by convection. The entire air inlet is located on one end wall of the barn with all of the exhaust fans located on the opposite end [2,10,76,92].

Chaiyabutr et al. [78], Smith et al. [80,81], and Shiao et al. [83] investigated the efficacy of using a tunnel-ventilated, water-padded free-stall barn to reduce heat stress in dairy cows (Table 7). Globally, this system improves environmental conditions and the performance of animals compared with open-sided barns alone or with fans and/or sprinklers. Actually, tunnel barns equipped with evaporative cooling pads reduced THI by up to 4.9 units, resulting in decreased rectal temperatures (0.4–1.0 °C) and respiration rates (2–22 breaths min^−1^) and increased dry matter intake (0.3–2.0 kg**∙**day^−1^) and milk yield (0.3–5.3 kg**∙**day^−1^). Bucklin et al. [32] tested a tunnel barn equipped with sprinklers. The conditions within the building were greatly improved (−5.9 THI units) compared with those outside and slightly better (−0.7 THI unit) relative to an open shed with fans and sprinklers (Table 10).

#### 3.1.4. Low-Profile, Cross-Ventilated Barns

Low-profile, cross-ventilated (LPCV) barns were developed to move air parallel to the body of the cows when they are lying in stalls while traditional tunnel ventilation moves air parallel to the ridge of the building. A ceiling could be used to limit the size of the cross sectional area. However, most often, vertical baffles are used to accelerate the air at cow level to the desired velocity [2,10,84,85,93,94,95]. In fact, baffles can increase air speed to 2.7–3.6 m∙s^−1^ from 0.9–1.3 m∙s^−1^ [96].

Smith et al. [82] assessed the effect of evaporative pads on core-body temperature and lying behaviour of lactating Holstein cows housed in LPCV free-stall facilities in a humid environment. They showed that this combination of systems reduced THI by 3.3 units, can decrease vaginal temperature (0.1 °C), and tended to increase lying time.

### 3.2. Sprinklers

Sprinklers are one of the most common and effective methods to promote heat loss [9]. They generate droplets that wet the cow’s hair coat and skin. Fans force air over the cow’s body, causing evaporative cooling to take place on its surface. Sprinklers use low-pressure nozzles (10–40 psi) with flow rates of about 0.75–2 L∙min^−1^ run for 1–3 min every 5–15 min in the 21–27 °C temperature range [2,10,22,58,59,85]. However, they are known to generate a large volume of water to be processed [9,59], about 10 times (200–530 and 2050–5350 L∙day^−1^) that of misting systems [71].

Sprinkling systems have been studied for many decades to establish their effect on cow performance (Table 10). Early works [97,98] established that cows show rapid changes toward normal body temperature and respiration rate when they were removed from sunshine after 2 h, hand-sprinkled with water, and then subjected to a gentle breeze produced by a fan for 1 h. The impact of spray cooling without fans was further assessed on dairy cows maintained under loose-housing conditions [33,42,99,100,101]. Rectal temperatures and respiration rates were either similar or lower for sprinkled cows when compared with those of shaded cows. The addition of a sprinkling system alone had opposing results on daily milk yield since it did not always allow for a production increase. The combination of sprinklers and fans in dairy barns was evaluated in different locations of the US, Argentina, Iran, Israel, and Italy [32,66,70,71,75,102,103,104,105,106,107,108,109,110]. In general, thermal environments were changed with the cooling system since temperature was reduced (0.2–4.9 °C) and relative humidity was increased (0.6–24.4 units of percentage), resulting in decreased THI (0.2–5.9 units). This result is in accordance with the formula developed by St-Pierre et al. [8] from published data which quantified similar declines in THI for sprinkler-fan systems used in hot conditions. Although spraying time differed between the studies (20 s to 30 min), sprinkler and fan cooling resulted in lower body temperatures (0.3–1.1 °C) and respiration rates (7–55 breaths min^−1^) and improved dry matter intake (0.9–3.0 kg**∙**day^−1^) and milk yield (1.0–4.1 kg**∙**day^−1^**∙**cow^−1^).

### 3.3. Conductive Cooling

Conductive cooling allows heat transfer from a direct contact between a cow lying down and a cooled surface (bedding, water mattress, or any other water-cooled heat exchanger embedded under the bedding) [111,112]. Little research has been done evaluating the effectiveness of conductive cooling on dairy cows, mainly because they only have 20% of their surface area available to exchange heat via conduction [113].

#### 3.3.1. Bedding

In commercial dairy farms, bedding material is selected based on economic feasibility, cow comfort, cleanliness, and udder health. However, few producers consider the thermal comfort that this bedding material provides. Cummins [114] suggests that the bedding material in a dairy farm should be part of their heat abatement strategies; lower temperatures were reported for limestone (25.9 °C) and sand (26.9 °C) compared with wood shavings (28.6 °C) at 25 mm below the surface. A more recent computational and experimental study showed that sand bedding had higher heat flux for cows when compared with straw and mattresses filled with rubber granules [115]. A study [116] was also conducted to evaluate the use of heat exchangers buried under 25 cm of bedding (sand or dried manure) as components in a conductive system for cooling cows. Sand bedding remained cooler than dried manure bedding in all environments (thermoneutral, hot and dry, and hot and humid) and at all levels of cooling (water on or off).

#### 3.3.2. Water-Cooled Heat Exchangers

Conductive cooling with water-cooled heat exchangers has the advantages of recycling water as a working fluid in a closed-loop system and requiring less energy than evaporative cooling units. This application may also improve animal hygiene and reduce humidity in the barn compared with evaporative cooling systems [112]. 

The first work involving the use of cooled waterbeds (water-filled compartment between two layers of rubber reinforced with synthetic fabric to add stability) included experimental and modelling parts [117]. The modelling component of the study involved a finite difference model which predicted that cows resting on waterbeds (water cooled at 10 °C) can lose about 20–25% of their metabolic heat (about 1600 W) by conduction. Based on computational fluid dynamics (CFD), Rojano et al. [118] found that a heat exchanger placed under the bedding contributes to 20% of the cooling effect under cool air conditions (18 °C), whereas under hot air conditions (38 °C) the contribution increases to nearly 90%. Subsequently, Mondaca et al. [119] developed a conjugate heat and mass transfer 2-D model which demonstrates that the portion of heat removed by conduction (20%) remains relatively constant at all temperatures when the air is dry and the cow’s skin is fully wetted. However, neither of the last two studies included experimental studies with live animals, and thus the models have not been fully validated. Perano et al. [120] conducted an extensive experimental study exposing animals to hot and humid conditions (THI of 79–81 units) and only half of the cows to cooled water mattresses (4.5 or 10 °C). When cows were conductively cooled with 4.5 °C water, rectal temperature, respiration rate, dry matter intake, and milk yield were respectively improved by 1.0 °C, 18 breaths min^−1^, 3.1 kg∙day^−1^, and 2.5 kg∙day^−1^ compared with control cows (Table 11). Rectal temperature was 0.3 °C lower when cows were cooled with 4.5 °C circulating water versus 10 °C circulating water, but other variables did not show a significant difference between the cooling water temperatures. Ortiz et al. [116] evaluated the effectiveness of conductive cooling of dairy cows with heat exchangers (water at 7 °C) buried under 25 cm of bedding (sand or dried manure). No significant difference in respiratory frequency between the cooled and the uncooled cows was reported, but the core-body temperature was respectively decreased by 0.13 °C and 0.14 °C when sand and dried manure were used as bedding. Recently, Gebremedhin et al. [112] developed a 3-D CFD model establishing that the heat flux for a water mattress at 4.6 °C was 430 W m^−2^ and dropped to 306 W m^−2^ for a water temperature of 14.5 °C.

## 4. Discussion

The available information unmistakably shows that heat stress negatively impacts cow performance, resulting in important economic losses for the dairy industry. The severity of heat stress issues will increase as global warming progresses, especially if genetic selection for growth rate and milk yield continues. Fortunately, major advances in environmental management, including cooling systems, can attenuate the effects of thermal stress on health, production, and reproduction [9]. Although several cooling strategies exist, their efficiency in humid continental climates is still unclear since experiments were mostly conducted under subtropical conditions [17].

The benefits of shades and roof insulation on thermal environment and cow performance through reducing the impact of solar radiation are undeniable no matter the climate zone (Table 1 and Table 2). Even in a temperate humid environment in New Zealand [30], some advantages were recorded on the BGHI value (−2 units) and milk production (+0.4 kg∙day^−1^) when shade was used. Besides keeping the barn cooler in summer, roof insulation can also reduce the flow of cold air in winter for northern farms [10,40]. Therefore, shading devices at pastures and roof insulation of barns should be used every time it is possible.

Typically, dairy barns in humid continental climates are naturally- or mechanically-ventilated, close-sided facilities. In such cases, air conditioning could be an option and likely the most effective way to reduce and maintain air temperature and relative humidity under an acceptable level where THI < 72 [32]. However, due to energy costs and system maintenance issues (e.g., dust filtration problems for recirculated air, odour-ammonia accumulation), air conditioning (including inspired-air cooling) has been recognised as cost-prohibitive, even in hot climates, and thus air-conditioned barns are uncommon today [17,52,55,61,121].

A combination of fans (to increase convective heat loss) and either sprinklers or foggers/misters (to promote evaporative cooling) has been shown to be the most effective way to cool dairy cows other than using air conditioning [90]. In fact, the addition of fans inside existing buildings to recirculate the air successfully reduced heat stress (Table 12) as shown by decreased body temperatures (−0.5 °C) and respiration rates (−11 breaths min^−1^), and enhanced milk production (+1.0 kg∙day^−1^). The levels of improvement of physiological responses by fans alone were near to those reached with evaporative cooling systems (−0.6 °C, −17 breaths min^−1^, and +2.0 kg∙day^−1^). Based on data in Table 9, it seems that LVHS fans are more efficient than HVLS fans. For new or remodelled constructions, tunnel-ventilated and LPCV barns (generally equipped with evaporative cooling systems in the reported literature) would also provide a desired chilling effect (Table 12). Nevertheless, the application of LPCV barns may be limited due to the minimum practical size of 400 cows and the few existing guidelines for operating fans during winter in northern countries [96].

High-pressure foggers and misters are known to be especially effective in dry regions in open-sided, ridge-vented, tall barns [2,10,22,58,59]. Therefore, they do not work well in humid environments in close-sided barns because fog or fine mists can cause a more humid microenvironment and inhibit cooling by reducing the vapour pressure potential [89,102,104]. Actually, increasing relative humidity by 10 to 20 units of percentage with the use of foggers or misters (Table 12) when the average summer relative humidity is already 75% in humid continental climates can eradicate any benefit from a potential air temperature drop. In addition, there is some suggestion that the environment produced by these evaporative cooling systems may predispose cows to respiratory disease, especially in enclosed areas [9,22,38,58,60]. Foggers and misters are also particularly prone to clogging and require regular maintenance and control [10,22,58,59,85].

For these reasons, low-pressure sprinklers are more common [122]. In fact, sprinkler-fan systems are among the best strategies to reduce thermal stress (THI decreases of 2.5 units) and to improve dairy cows performance and welfare (−0.5 °C of rectal temperature, −22 breaths min^−1^, and +2.5 kg**∙**day^−1^ of milk) with only a moderate increase in relative humidity (Table 12). This is why Flamenbaum et al. [102] and Strickland et al. [104] suggest using sprinklers with large droplet sizes as opposed to foggers and misters to achieve effective cooling in humid regions. Moreover, some mechanically-ventilated facilities in humid subtropical climates rely on both evaporative cooling pads to cool the air and low-pressure sprinklers to further cool the cows at the feed-line [122]. Even in high humidity environments created by the evaporative cooling systems, feed-line soaking would provide additional cooling for the cattle [61]. However, based on the results of Shiao et al. [83] in Taiwan, the combination of evaporative cooling, feed-line soaking, and tunnel ventilation did not really change cow physiological responses and increased temperature and THI (Table 7). Therefore, the use of sprinklers and/or evaporative cooling pads could negatively affect environmental conditions in humid continental climates (e.g., pads increase relative humidity by 24 units of percentage; Table 12).

Furthermore, water-based options such as sprinkler-fan systems and evaporative cooling pads may require a significant amount of water and electricity [119] and careful design, operation, and regular maintenance in order to make them work effectively [76]. A direct-contact conductive cooling method that circulates chilled water or low-temperature groundwater in mattresses or heat exchangers may provide a more effective system in terms of energy and water. Indeed, the water, after passing through the system, could be cycled through a refrigeration unit and then reused [112,119]. Such a system enhances heat loss and thereby cools the animal even under hot and humid conditions (Table 11). Additionally, waterbeds can provide benefits such as reduction of lesions, swollen hocks, and cull rates as well as stall cleanliness [123].

## 5. Conclusions and Implications

The selection of cooling systems to improve cow health and performance without worsening ambient conditions under a humid continental climate can differ from areas that are more arid. Options limiting the flow of heat into the barn or allowing increased convection or conduction should be preferred. In that sense, shade, roof insulation, circulation fans, tunnel ventilation, and water-cooled mattresses are the practical cooling strategies that should be considered under a humid continental climate. All these applications could be implemented on new or remodelled dairy facilities. The installation of evaporative cooling systems such as pads, misters, and sprinklers is not recommended in environments where relative humidity could reach over 75% due to the increase in humidity associated with these systems. The massive need for water resources of evaporative cooling options could also be an issue. Determining practices that will optimize the combination of increased air velocity and conductive cooling in terms of milk yield, reproduction performances, animal health, and resource efficiency in humid continental climates will require more research, especially from a climate change perspective, as global temperature will continue to rise. In this context, while countries with advanced dairy production systems are making many efforts to enhance animal welfare by providing the best environment possible in terms of housing conditions, heat stress is often neglected since it is considered outside of the farmers’ control. On an ethical point of view, the implementation of heat stress control strategies that can improve cow well-being by lessening their body temperature and respiration rate should be as important as the addition of bedding to ensure cow comfort. Thus, future initiatives, programs, or standards developed to meet societal expectations related to animal care, health, welfare as well as environmental stewardship (e.g., proAction in Canada) should include extreme heat mitigation as a key point. The information contained herein should help to point out the best options in humid continental dairy farms.

## Figures and Tables

**Table 1:** Meteorological and thermal stress attributes and physiological response for non-shaded (NS) and shaded (S) environments.

Reference	Location	Breed	System	T_db_	RH ^1^	T_dp_^2^	WS	T_bg_	THI ^3^	BGHI ^4^	HLI ^5^	BT ^6^	RR	MY
				(°C)	(%)	(°C)	(m∙s^−1^)	(°C)				(°C)	(Breaths min^−1^)	(kg∙day^−1^)
Collier et al. [24]	Florida, US	Holstein, Jersey	NS									39.6	115	
			S									38.7	79	
Muller et al. [25]	South Africa	Friesian	NS											19.0
			S											20.1
Roman-Ponce et al. [26]	Florida, US	Holstein, Jersey, Guernsey, Brown Swiss	NS	28	78	24	2	37	79	87	96	39.4	82	15.0
			S	28	79	24	2	28	79	78	83	38.9	54	16.6
Schütz et al. [27]	New Zealand	Holstein-Friesian	NS	20	58	11	11	24	65	70	62			
			S	20	64	13		22	66	68				
Schütz et al. [28]	New Zealand	Holstein-Friesian	NS	27	42	13	7	29	73	75	74			
			S	25	49	14		26	71	72				
Schütz et al. [29]	New Zealand	Holstein-Friesian	NS	22	59	14	6	30	69	76	78	38.5	62	18.8
			S	23	61	15		24	69	71		38.4	54	18.4
Fisher et al. [30]	New Zealand	Holstein-Friesian	NS	18	80	14		21	63	68		39.0		13.9
			S	19	77	15		19	64	66		38.9		14.3
Kendall et al. [33]	New Zealand	Holstein-Friesian	NS									38.9	78	13.2
			S									38.6	54	14.0
Tucker et al. [34]	New Zealand	Holstein-Friesian	NS									37.9		
			S									37.7		
Muller et al. [35]	South Africa	Friesian	NS									38.9	78	
			S									38.6	64	
Kendall et al. [36]	New Zealand	Holstein-Friesian	NS									38.9		17.2
			S									38.6		17.7

Abbreviations: T_db_ = dry-bulb temperature, RH = relative humidity, T_dp_ = dew-point temperature, WS = wind speed, T_bg_ = black globe temperature, THI = temperature-humidity index, BGHI = black globe-humidity index, HLI = heat load index, BT = body temperature, RR = respiration rate, MY = milk yield; ^1^ Calculated from Lawrence [43] if not given by the authors: RH = 100 × EXP[−5419(T_db_ − T_dp_)/(T_db_ + 273.15)/(T_dp_ + 273.15)]; ^2^ Calculated from Lawrence [43] if not given by the authors: T_dp_ = T_db_ − [((100 − RH)/5)((T_db_ + 273.15)/300)^2^] − 0.00135(RH − 84)^2^ + 0.35; ^3^ Calculated from Schüller et al. [44] if not given by the authors: THI = (1.8T_db_ + 32) − [(0.55 − 0.0055RH)(1.8T_db_ − 26)]; ^4^ Calculated from Buffington et al. [45]: BGHI = T_bg_ + 0.36T_dp_ + 41.5; ^5^ Calculated from Gaughan et al. [46] if not given by the authors: HLI = IF[T_bg_ > 25, 8.62 + (0.38RH)(1.55 T_bg_) + EXP(2.4 − WS) − 0.5WS, 10.66 + (0.28RH) + (1.3T_bg_) − WS]; ^6^ Body temperature refers to rectal temperature for studies in Florida and South Africa and to vaginal temperature for studies in New Zealand.

**Table 2:** Meteorological and thermal stress attributes and physiological response of dairy cows for non-insulated (NI) and insulated (I) shades.

Reference	Location	System	T_db_	RH	T_dp_^1^	T_bg_	THI ^2^	BGHI ^3^	DMI	MY
			(°C)	(%)	(°C)	(°C)			(kg∙day^−1^)	(kg∙day^−1^)
Daniel et al. [41]	Mississippi, US	NI							18.2	17.9
		I							18.4	18.9
Fuquay et al. [42]	Mississippi, US	NI	31.7	56.4	22.0	32.8	81.6	82.2		24.1
		I	30.5	58.2	21.4	30.8	80.3	80.0		24.3

Abbreviations: T_db_ = dry-bulb temperature, RH = relative humidity, T_dp_ = dew-point temperature, T_bg_ = black globe temperature, THI = temperature-humidity index, BGHI = black globe-humidity index, DMI = dry matter intake, MY = milk yield; ^1^ Calculated from Lawrence [43]: T_dp_ = T_db_ − [((100 − RH)/5)((T_db_ + 273.15)/300)^2^] − 0.00135(RH − 84)^2^ + 0.35; ^2^ Calculated from Schüller et al. [44]: THI = (1.8T_db_ + 32) − [(0.55 − 0.0055RH)(1.8T_db_ − 26)]; ^3^ Calculated from Buffington et al. [45]: BGHI = T_bg_ + 0.36T_dp_ + 41.5.

**Table 3:** Meteorological attributes and THI calculations for control treatments and air-conditioned dairy barns.

Reference	Location	Breed	System	T_db_	RH	THI ^1^
				(°C)	(%)	
Bucklin et al. [32]	Florida, US	Holstein	Outside	27.7	70.0	77.9
			Air-conditioned barn	22.0	85.0	70.5
Hahn et al. [52]—Year 1	Missouri, US	Holstein	Dry lot	25.9	66.9	74.9
			Air-conditioned barn	23.4	65.2	71.0
Hahn et al. [52]—Year 2	Missouri, US	Holstein	Dry lot	23.4	73.8	71.8
			Air-conditioned barn	23.2	67.0	70.9

Abbreviations: T_db_ = dry-bulb temperature, RH = relative humidity, THI = temperature-humidity index; ^1^ Calculated from Schüller et al. [44]: THI = (1.8T_db_ + 32) − [(0.55 − 0.0055RH)(1.8T_db_ − 26)].

**Table 4:** Meteorological attributes, THI calculations, and physiological response of dairy cows in shaded environments (S) with or without zone cooling systems (ZC).

Reference	Location	Breed	System	T_db_	RH	THI ^1^	RT	RR	MY
				(°C)	(%)		(°C)	(Breaths min^−1^)	(kg∙day^−1^)
Canton et al. [31]	Florida, US	Holstein-Friesian	S				38.9	77	
			S + ZC				38.6	62	
Fuquay et al. [42]—Year 1	Mississippi, US	Unknown	S	31.7	56.4	81.6			
			S + ZC	26.9	71.1	76.9			
Fuquay et al. [42]—Year 2	Mississippi, US	Unknown	S	29.8	54.5	78.7	38.8	78	
			S + ZC	26.5	65.2	75.5	38.7	71	
Roussel and Beatty [56]	Louisiana, US	Holstein, Jersey, Guernsey	S				40.0	87	17.3
			S + ZC				39.4	82	20.6
Gomila et al. [57]	Louisiana, US	Holstein, Guernsey	S				39.1	77	19.7
			S + ZC				38.9	64	21.0

Abbreviations: T_db_ = dry-bulb temperature, RH = relative humidity, THI = temperature-humidity index, RT = rectal temperature, RR = respiration rate, MY = milk yield; ^1^ Calculated from Schüller et al. [44]: THI = (1.8T_db_ + 32) − [(0.55 − 0.0055RH)(1.8T_db_ − 26)].

**Table 5:** Meteorological attributes and THI calculations between different housing systems for dairy cows in the study of Bucklin et al. [32].

System	T_db_	RH	THI ^1^
	(°C)	(%)	
Outside	27.5	84	79.4
Tunnel barn with low-pressure feed faced sprinklers	27.8	89	80.6
Tunnel barn with high-pressure foggers and low-pressure feed faced sprinklers	25.7	99	78.1

Abbreviations: T_db_ = dry-bulb temperature, RH = relative humidity, THI = temperature-humidity index; ^1^ Calculated from Schüller et al. [44]: THI = (1.8T_db_ + 32) − [(0.55 − 0.0055RH)(1.8T_db_ − 26)].

**Table 6:** Meteorological attributes, THI calculations, and physiological response of dairy cows for control treatments and barns equipped with mist-fan systems.

Reference	Location	Breed	System ^1^	T_db_	RH	THI ^2^	BT ^3^	RR	DMI	MY
				(°C)	(%)		(°C)	(Breaths min^−1^)	(kg∙day^−1^)	(kg∙day^−1^)
Brouk et al. [66]	Kansas, US	Holstein	UB	33.3	36.0	80.0	40.2	115		
			UB + M + F	24.7	85.5	74.8	39.1	67		
Boonsanit et al. [67]	Thailand	Crossbred Holstein	OB	31.0	70.1	82.8	39.4	70		
			OB + M + F	29.2	78.4	81.4	38.9	58		
Calegari et al. [68]	Italy	Friesian	OB + F					60		29.8
			OB + M + F					56		30.8
Correa-Calderon et al. [70]	Arizona, US	Holstein	S				39.7	87		
			S + M + F				38.8	50		
Correa-Calderon et al. [70]	Arizona, US	Brown Swiss	S				39.2	79		
			S + M + F				38.8	52		
Lin et al. [71]—Year 1	Alabama, US	Holstein	OB + F	27.9	68.3	78.0		66	17.1	22.4
			OB + M + F	25.9	81.0	76.5		57	18.5	23.0
Lin et al. [71]—Year 2	Alabama, US	Holstein	OB + F	31.5	69.5	83.6		77	17.5	21.0
			OB + M + F	26.6	88.5	78.5		66	19.6	24.2
Takamitsu et al. [72]—Test 1	Japan	Holstein	OB				40.1	69	8.7	18.4
			OB + M + F				39.3	53	9.4	20.3
Takamitsu et al. [72]—Test 2	Japan	Holstein	OB				39.8	69	8.5	18.5
			OB + M + F				38.8	54	9.1	19.6
Tarazón-Herrera et al. [73]	Arizona, US	Holstein	S	38.0	26.1	83.2	39.5	84	23.4	27.7
			S + M + F	32.3	36.6	78.9	38.7	67	23.4	29.0
Frazzi et al. [74]	Italy	Friesian	OB				39.5	94		29.0
			OB + M + F				38.6	70		29.4
Frazzi et al. [75]	Italy	Friesian	OB						21.8	30.6
			OB + M + F						22.1	34.8

Abbreviations: T_db_ = dry-bulb temperature, RH = relative humidity, THI = temperature-humidity index, BT = body temperature, RR = respiration rate, DMI = dry matter intake, MY = milk yield; ^1^ Housing system components: UB = unknown type of barn, M = mister, F = circulation fan, OB = open-sided barn, S = shade; ^2^ Calculated from Schüller et al. [44] if not given by the authors: THI = (1.8T_db_ + 32) − [(0.55 − 0.0055RH)(1.8T_db_ − 26)]; ^3^ Body temperature refers to rectal temperature for all studies except for Brouk et al. [66] and Takamitsu et al. [72] (first experimental test) who used vaginal temperature.

**Table 7:** Meteorological attributes, THI calculations, and physiological response of dairy cows for control treatments and barns equipped with evaporative cooling pads.

Reference	Location	Breed	System ^1^	T_db_	RH	THI ^2^	BT ^3^	RR	DMI	MY
				(°C)	(%)		(°C)	(Breaths min^−1^)	(kg∙day^−1^)	(kg∙day^−1^)
Brown et al. [77]—Year 1	Mississippi, US	Unknown	OB + F				40.1	95		18.9
			OB + P				39.8	91		20.5
Brown et al. [77]—Year 2	Mississippi, US	Unknown	OB + F				39.1	78		24.9
			OB + P				39.0	74		25.4
Brown et al. [77]—Year 3	Mississippi, US	Unknown	OB + F				38.9	65		22.5
			OB + P				38.8	64		21.4
Chaiyabutr et al. [78]	Thailand	Crossbred Holstein	OB	33.4	61	84.8	39.7	86		10.8
			TVB + P	27.8	84	79.9	38.7	64		16.1
Chen et al. [79]	Arizona, US	Holstein	S				39.1	82		27.5
			S + P				38.6	64		30.0
Smith et al. [80,81]—Year 1	Mississippi, US	Holstein	OB + K + F	33.1	64.4	85.0	39.0	68	14.8	22.5
			TVB + P	30.1	91.1	84.8	38.6	55	16.5	25.1
Smith et al. [80,81]—Year 2	Mississippi, US	Holstein	OB + F	24.9	71.3	73.8	39.3	71	14.2	26.7
			TVB + P	22.6	93.5	72.2	38.7	55	16.2	29.5
Smith et al. [82]	Minnesota, US	Holstein	O	25.4	64.3	73.8				
			CVB	25.4	62.7	73.7	38.7			
			CVB + P	21.9	86.0	70.4	38.6			
Shiao et al. [83]	Taiwan	Holstein	OB + K + F	30.9	75.7	83.7	38.9	54	18.5	24.7
			TVB + P	27.1	99.9	80.8	39.1	52	18.8	25.0
			TVB + P + K	27.7	98.8	81.7	38.9	56	18.8	25.4

Abbreviations: T_db_ = dry-bulb temperature, RH = relative humidity, THI = temperature-humidity index, BT = body temperature, RR = respiration rate, DMI = dry matter intake, MY = milk yield; ^1^ Housing system components: OB = open-sided barn, F = circulation fan, P = evaporative cooling pad, S = shade, K = sprinkler, TVB = tunnel-ventilated barn, O = outside, CVB = cross-ventilated barn; ^2^ Calculated from Schüller et al. [44]: THI = (1.8T_db_ + 32) − [(0.55 − 0.0055RH)(1.8T_db_ − 26)]; ^3^ Body temperature refers to rectal [78,79,80,81] or vaginal [82,83] temperature. In one case [77], BT location was unknown.

**Table 8:** Physiological response of dairy cows for control treatments and barns equipped with high-speed fans.

Reference	Location	Breed	System ^1^	BT ^2^	RR	CR	DMI	MY
				(°C)	(Breaths min^−1^)	(%)	(kg∙day^−1^)	(kg∙day^−1^)
Berman et al. [6]	Israel	Holstein	S		73			
			S + F		59			
Takamitsu et al. [72]	Japan	Holstein	OB	40.1	69		8.7	18.4
			OB + F	39.6	59		9.3	19.4
Frazzi et al. [74]	Italy	Friesian	OB	39.5	94			29.0
			OB + F	38.9	78			29.6
Folman et al. [86]	Israel	Friesian	OB	39.8		22		34.3
			OB + F	39.3		52		36.2
Calegari et al. [87]	Italy	Friesian	OB + K	38.8	56			32.1
			OB + K+ F	38.7	54			32.4

Abbreviations: BT = body temperature, RR = respiration rate, CR = conception rate, DMI = dry matter intake, MY = milk yield; ^1^ Housing system components: S = shade, F = circulation fan, OB = open-sided barn, K = sprinkler; ^2^ Body temperature refers to rectal temperature for all studies except for Takamitsu et al. [72] who used vaginal temperature.

**Table 9:** Meteorological attributes, THI calculations, and physiological response of dairy cows for control treatments and barns equipped with ceiling fans.

Reference	Location	Breed	System ^1^	T_db_	RH	THI ^2^	VT	RR	MY
				(°C)	(%)		(°C)	(Breaths min^−1^)	(kg∙day^−1^)
Bucklin et al. [32]	Florida, US	Holstein	O	33.9	50.0	83.4			
			OB + K + CF	29.7	55.0	78.7			
Worley and Bernard [90]	Georgia, US	Holstein	OB + M + F				39.3		
			OB + CF				39.5		
Meyer et al. [91]	Kansas, US	Holstein	OB + K + F					75	40.1
			OB + K + CF					84	37.1

Abbreviations: T_db_ = dry-bulb temperature, RH = relative humidity, THI = temperature-humidity index, VT = vaginal temperature, RR = respiration rate, MY = milk yield; ^1^ Housing system components: O = outside, OB = open-sided barn, K = sprinkler, CF = ceiling fan, M = mister, F = circulation fan; ^2^ Calculated from Schüller et al. [44]: THI = (1.8T_db_ + 32) − [(0.55 − 0.0055RH)(1.8T_db_ − 26)].

**Table 10:** Meteorological attributes, THI calculations, and physiological response of dairy cows for control treatments and barns equipped with sprinklers.

Reference	Location	Breed	System ^1^	T_db_	RH	THI ^2^	BT ^3^	RR	DMI	MY
				(°C)	(%)		(°C)	(Breaths min^−1^)	(kg∙day^−1^)	(kg∙day^−1^)
Bucklin et al. [32]	Florida, US	Holstein	O	33.9	50	83.8				
			OB + K + F	29.7	55	78.6				
			TVB + K	29.0	56	77.9				
Kendall et al. [33]	New Zealand	Holstein-Friesian	S				38.6	54		14.0
			S + K				38.6	24		13.6
Fuquay et al. [42]	Mississippi, US	Unknown	S	30.5	60.1	80.6	38.9	84		
			S + K	29.7	64.0	80.0	38.8	82		
Brouk et al. [66]	Kansas, US	Holstein	UB				40.2	115		
			UB + K + F				39.1	60		
Correa-Calderon et al. [70]	Arizona, US	Holstein	S				39.7	87		
			S + K + F				39.0	64		
Correa-Calderon et al. [70]	Arizona, US	Brown Swiss	S				39.2	79		
			S + K + F				38.9	61		
Lin et al. [71]—Year 1	Alabama, US	Holstein	OB + F	27.9	68.3	78.0		66	17.1	22.4
			OB + K + F	25.5	84.3	76.2		54	19.0	25.0
Lin et al. [71]—Year 2	Alabama, US	Holstein	OB + F	31.5	69.5	83.6		77	17.5	21.0
			OB + K + F	27.5	93.9	80.7		58	20.5	24.0
Frazzi et al. [75]	Italy	Italian Friesian	OB						21.8	30.6
			OB + K + F						22.7	33.5
Seath and Miller [97]	Louisiana, US	Jersey	O				39.3	83		
			S				38.8	58		
			S + K				38.7	41		
Seath and Miller [98]	Louisiana, US	Jersey	O				39.7	109		
			CB				39.3	88		
			CB + K				39.0	77		
			CB + F				38.9	72		
			CB + K + F				38.7	74		
Igono et al. [99]	Missouri, US	Holstein	S				39.1			23.2
			S + K				38.8			23.9
Chen et al. [100]	California, US	Holstein-Friesian	OB				38.9			42.6
			OB + K				38.6			46.1
Flamenbaum et al. [102]	Israel	Holstein	OB				38.9			
			OB + K + F				38.2			
Igono et al. [103]	Missouri, US	Holstein	S	31.0	43.0	78.5	39.2		32.8 ^4^	23.3
			S + K + F	30.8	43.6	78.3	38.8		35.1 ^4^	25.3
Strickland et al. [104]	Florida, US	Holstein	OB					95	17.8	18.1
			OB + K + F					57	19.1	20.2
Her et al. [105]	Israel	Holstein	OB				39.2			
			OB + K + F				38.6			
Valtorta and Gallardo [106]	Argentina	Holstein	S				39.5	72		22.1
			S + K + F				39.2	54		23.2
Keister et al. [107]—Year 1	Arizona, US	Jersey	S							21.8
			S + K + F							23.1
Keister et al. [107]—Year 2	Arizona, US	Jersey	S	32.8	40.3	80.2		102		20.5
			S + K + F	29.1	59.3	78.5		80		22.4
Karimi et al. [108]	Iran	Holstein	OB				39.5	70	13.7	40.5
			OB + K + F				39.2	63	15.5	44.6
Hillman et al. [109]	New York, US	Holstein	UB				40.0	90		
			UB + K + F				39.3	74		
Turner et al. [110]	Kentucky, US	Holstein	OB				39.2	91	34.9 ^4^	22.7
			OB + K + F				38.7	75	38.1 ^4^	26.3

Abbreviations: T_db_ = dry-bulb temperature, RH = relative humidity, THI = temperature-humidity index, BT = body temperature, RR = respiration rate, DMI = dry matter intake, MY = milk yield; ^1^ Housing system components: O = outside, OB = open-sided barn, K = sprinkler, F = circulation fan, TVB = tunnel-ventilated barn, S = shade, UB = unknown type of barn, CB = close-sided barn; ^2^ Calculated from Schüller et al. [44]: THI = (1.8T_db_ + 32) − [(0.55 − 0.0055RH)(1.8T_db_ − 26)]; ^3^ Body temperature refers to rectal temperature in all studies except those of Kendall et al. [33], Brouk et al. [66], and Chen et al. [100], which used vaginal temperature; ^4^ Feed intake (kg∙day^−1^, as fed).

**Table 11:** Physiological response of dairy cows for a barn equipped with waterbeds at different water temperatures.

Reference	Location	Breed	System	RT	RR	DMI	MY
				(°C)	(Breaths min^−1^)	(kg∙day^−1^)	(kg∙day^−1^)
Perano et al. [120]	New York, US	Holstein	Uncooled	40.1	82	18.9	30.3
			Cooled at 10 °C	39.4	68	20.9	32.6
			Cooled at 4.5 °C	39.1	64	22.0	32.8

Abbreviations: RT = rectal temperature, RR = respiration rate, DMI = dry matter intake, MY = milk yield.

**Table 12:** Average improvements of potential cooling systems for humid continental farms on meteorological and thermal stress attributes and physiological response of dairy cows.

Method	Cooling System	T_db_	T_bg_	RH	THI	BGHI	HLI	RT	VT	RR	CR	FI	DMI	MY
		(°C)	(°C)	(%)				(°C)	(°C)	(Breaths min^−1^)	(%)	(kg∙day^−1^)	(kg∙day^−1^)	(kg∙day^−1^)
Modifying the environment to limit the degree of heat stress	Shade	0.0	−4.4		0.0	−4.2	−13.0	−0.6	−0.2	−22				+0.7
	Roof insulation	−1.2	−2.0		−1.3	−2.2							+0.2	+0.6
	Evaporative cooling pad	−3.5		+23.7	−2.4			−0.6	0.0	−10			+1.3	+1.9
	Fogger	−2.1		+10.0	−2.5									
	Mister-fan	−4.6		+20.0	−3.5			−0.8	−1.0	−20			+0.9	+1.7
Enhancing heat exchange between cows and their environment	Sprinkler	−0.8		+3.9	−0.6			−0.2	−0.1	−16				+1.3
	Sprinkler-fan	−2.8		+11.1	−2.5			−0.5	−1.1	−22		+2.8	+1.8	+2.5
	Tunnel ventilation (+pad)	−3.5		+23.8	−2.2			−0.7	0.0	−12			+1.3	+2.9
	Cross ventilation (+pad)	−3.5		+21.7	−3.4									
	LVHS							−0.4	−0.5	−11	+30		+0.6	+1.0
	HVLS (+sprinkler)	−4.2		+5.0	−4.7									
	Waterbeds							−0.9		−16			+2.6	+2.4

Abbreviations: T_db_ = dry-bulb temperature, RH = relative humidity, T_bg_ = black globe temperature, THI = temperature-humidity index, BGHI = black globe-humidity index, HLI = heat load index, RT = rectal temperature, VT = vaginal temperature, RR = respiration rate, CR = conception rate, FI = feed intake, DMI = dry matter intake, MY = milk yield, LVHS = low volume, high speed fans, HVLS = high volume, low speed fans.
